# Whole-Exome Sequencing in Congenital Hypothyroidism Due to Thyroid Dysgenesis

**DOI:** 10.1089/thy.2021.0597

**Published:** 2022-05-17

**Authors:** Stéphanie Larrivée-Vanier, Martineau Jean-Louis, Fabien Magne, Helen Bui, Guy A. Rouleau, Dan Spiegelman, Mark E. Samuels, Zoha Kibar, Guy Van Vliet, Johnny Deladoëy

**Affiliations:** ^1^ Research Center of Centre Hospitalier Universitaire Sainte-Justine, Université de Montréal, Montréal, Canada.; ^2^ Department of Biochemistry, Université de Montréal, Montréal, Canada.; ^3^ Department of Endocrinology, McGill University Health Center, Montréal, Canada.; ^4^ Montreal Neurological Institute, McGill University, Montréal, Canada.; ^5^ Department of Medicine, Université de Montréal, Montréal, Canada.; ^6^ Department of Neurosciences, Université de Montréal, Montréal, Canada.; ^7^ Department of Pediatrics, Université de Montréal, Montréal, Canada.; ^8^ Pediatric Institute of Southern Switzerland, Bellinzona, Switzerland.; ^9^ Faculty of Biomedical Sciences, University of Southern Switzerland, Lugano, Switzerland.

**Keywords:** athyreosis, birth defects, nonsyndromic congenital hypothyroidism, thyroid dysgenesis, thyroid ectopy

## Abstract

**Context:**

Congenital hypothyroidism due to thyroid dysgenesis (CHTD) is a predominantly sporadic and nonsyndromic (NS) condition of unknown etiology. NS-CHTD shows a 40-fold increase in relative risk among first-degree relatives (1 in 100 compared with a birth prevalence of 1 in 4000 in the general population), but a discordance rate between monozygotic (MZ) twins of 92%. This suggests a two-hit mechanism, combining a genetic predisposition (incomplete penetrance of inherited variants) with postzygotic events (accounting for MZ twin discordance).

**Objective:**

To evaluate whether whole-exome sequencing (WES) allows to identify new predisposing genes in NS-CHTD.

**Methods:**

We performed a case–control study by comparing the whole exome of 36 nonconsanguineous cases of NS-CHTD (33 with lingual thyroid ectopy and 3 with athyreosis, based on technetium pertechnetate scintigraphy at diagnosis) with that of 301 unaffected controls to assess for enrichment in rare protein-altering variants. We performed an unbiased approach using a gene-based burden with a false discovery rate correction. Moreover, we identified all rare pathogenic and likely pathogenic variants, based on *in silico* prediction tools, in 27 genes previously associated with congenital hypothyroidism (CH) (thyroid dysgenesis [TD] and dyshormonogenesis).

**Results:**

After correction for multiple testing, no enrichment in rare protein-altering variants was observed in NS-CHTD. Pathogenic or likely pathogenic variants (21 variants in 12 CH genes) were identified in 42% of cases. Eight percent of cases had variants in more than one gene (oligogenic group); these were not more severely affected than monogenic cases. Moreover, cases with protein-altering variants in dyshormonogenesis-related genes were not more severely affected than those without.

**Conclusions:**

No new predisposing genes were identified following an unbiased analysis of WES data in a well-characterized NS-CHTD cohort. Nonetheless, the discovery rate of rare pathogenic or likely pathogenic variants was 42%. Eight percent of the cases harbored multiple variants in genes associated with TD or dyshormonogenesis, but these variants did not explain the variability of hypothyroidism observed in dysgenesis. WES did not identify a genetic cause in NS-CHTD cases, confirming the complex etiology of this disease. Additional studies in larger cohorts and/or novel discovery approaches are required.

## Introduction

Congenital hypothyroidism (CH) due to thyroid dysgenesis (TD) occurs in 1 of 4000 newborns (1,2). Congenital hypothyroidism due to thyroid dysgenesis (CHTD) results from a failure of the thyroid precursor cells to differentiate, to survive, or to migrate from the primordial pharynx to the neck (3). This results in either the absence of thyroid follicular cells (athyreosis) or, more commonly, in lingual thyroid ectopy (2). CHTD generally has no identified cause, and its incidence is not affected by any known environmental factor (1,2).

CHTD shows a female-to-male ratio of 3:1 (4). It is predominantly sporadic (98% of cases are nonfamilial) (5) and has a discordance rate of 92% between monozygotic (MZ) twins (6); these observations argue against fully penetrant classical Mendelian inheritance of CHTD. On the contrary, the occurrence of familial cases and the variation of the incidence by ethnicity suggest a genetic predisposition to CHTD. Indeed, the likelihood of CHTD in a first-degree relative is 1%, 40 times greater than in the general population (1/100 vs. 1/4000). In addition, CHTD is much less common in black African individuals (7). Lastly, several single-gene mutations (in *TSHR*, *NKX2.1*, *PAX8*, *FOXE1*, *GLIS3*, *NTN1*, *JAG1*, *CDCA8/BOREALIN*, and *TUBB1* (8–20)) cause CHTD, mainly orthotopic thyroid hypoplasia, most with associated syndromic features.

More than 95% of cases of CHTD remain unexplained, especially those with thyroid ectopy (8). This led us to propose a two-hit model to explain nonsyndromic (NS)-CHTD (21): first, a predisposing variant in the germ line is inherited or occurs *de novo*; second, somatic mechanisms might be involved, although our investigations into these mechanisms have been unsuccessful (21–25).

Recently, an enrichment in inherited protein-truncating variants has been reported in NS congenital heart disease (26). More generally, inherited variants might contribute to the phenotype of congenital malformations, although how this inherited predisposition leads to a phenotype remains to be further investigated. Sifrim *et al.* (26) also suggested that different study designs must be considered for the assessment of congenital heart disorders: a trio approach for the syndromic forms and a case–control approach for the NS ones. This prompted us to evaluate, for the first time, the burden of rare protein-altering variants in 36 NS-CHTD cases compared with 301 unaffected controls.

## Subjects and Methods

### Ethics statement

This study was approved by the Sainte Justine Ethics committee (ERB no. 94). All the parents or legal guardians provided written informed consent.

### Cohort and sample collection

Whole-exome sequencing (WES) of 30 cases with scintigraphy-proven TD was performed. WES data of six patients that have already been published (23,24) were reanalyzed in this study. All cases have NS-CHTD (33 ectopy and 3 true athyreosis) and they are mostly females (26 cases), as expected for CHTD.

The eligibility criteria were CH due to TD proven by scintigraphy and absence of other congenital anomalies. Three discordant MZ twins and one family in which both mother and daughter have ectopy were included. The control cohort consisted of 310 subjects without CH, whose exomes were produced from a next-generation sequencing (NGS) platform (Réseau de Médecine Génétique Appliquée). Controls were selected based on the absence of any endocrine disease, on a matched capture library kit, and on being unrelated to the NS-CHTD subjects. Exome data of both patients and controls were obtained using similar sequencing technologies and were analyzed through the same bioinformatic pipeline. Blood was obtained by peripheral venipuncture. DNA was extracted using standard methods and conserved at −20°C.

### Whole-exome sequencing

The cases and controls were sequenced using the Agilent 50 Mb SureSelect all exon V5 capture library, followed by Illumina base pair (2 × 100 bp) HiSeq 2000 or 2500 sequencing technology, as described previously (23,27). Cases were sequenced at Génome Québec (Montreal, Canada) and controls at Macrogen (Seoul, South Korea). In our experience, it is essential to match cases and controls not just for combined bioinformatic analysis, but also for the specific version of the exon capture kit obtained from the supplier. Different kits include different contents in both well-documented and newly annotated genes (especially nonprotein-coding genes such as lncRNAs).

### Bioinformatics

To acquire high-quality, single-nucleotide variant (SNV) data, the NGS raw fastQ data were cleaned by the Trimmomatic tools (28), aligned to the human reference genome (GRCh37.1/Hg19) using the Burrows–Wheeler Aligner with Maximal Exact Matches genome alignment tool, and the SNVs were called using the Genome Analysis Toolkit haplotype caller (29,30). The detailed SNV calling protocol of the Broad Institute SNV calling best practice pipeline was used (29). Our SNV called data set was annotated by ANNOVAR (31,32). The SNV annotation was done only for good-quality reads (total read depth >10 and variant read depth >5).

**FIG. 1.**
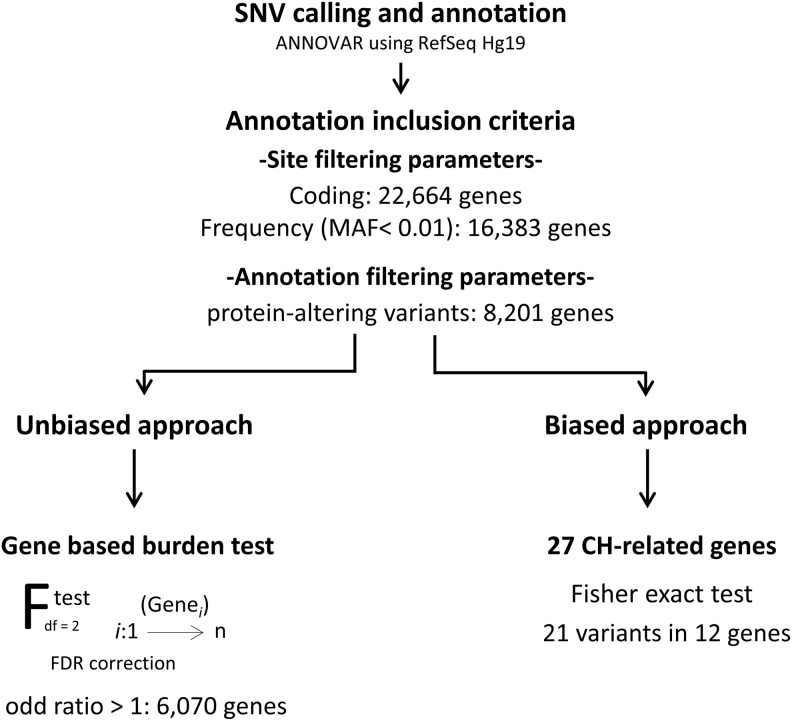
Analysis pipeline for whole-exome sequencing data of nonsyndromic-CHTD cases and controls. CHTD, congenital hypothyroidism due to thyroid dysgenesis.

The SNV data set was filtered to keep only high-quality rare SNVs with a probably damaging effect. The filtering criteria were as follows: (1) SNVs in a coding region or in exon–intron junctions, (2) with a minor allele frequency (MAF) ≤0.01 compared with the ExAC and GnomAD databases, (3) uncommon in the control data set (fewer than 5 controls with the SNV, fewer than 10 alleles per SNV in the control data set), (4) and with a presumably protein-altering effect (stop gain, frameshift, missense, or exon-adjacent intronic splice variants). A total of 8201 genes with potentially protein-altering variants passed all these criteria (Fig. 1).

Data manipulation was implemented using Python-based scripts version 3.5, and statistical modules such as Numerical Python and Scientific Python were used to build up the statistical functions of the scripts (33). Further statistical computation and graphics were processed with R scripts and R graphic packages (version 4.1.0) (34,35).

### Principal component analysis

Before the gene-based burden analysis, we performed a principal component analysis (PCA) to keep only controls with a similar ethnic background to that of the cases. First, we compared cases and controls data with the 1000 Genome (1KG) data. All cases and most controls clustered with Caucasian and admixed American samples according to the 1KG data (Supplementary Fig. S1). Next, we performed a PCA of our cases and controls to remove outliers by ancestry stratification. Nine controls, who were close to Asian or African 1KG samples, were removed from the 310 samples, resulting in 301 controls included in the gene burden analysis (Supplementary Fig. S1).

### Gene burden analysis

We performed a case–control study. The burden of rare potentially protein-altering variants for each gene was compared between the 36 NS-CHTD cases and the 301 controls. For each gene, we computed the frequency of the mutated genes observed in cases and controls followed by a one-tailed Fisher exact test. A false discovery rate (FDR) correction on gene-associated *p*-values that had an odds ratio >1 was applied, based on guidelines from the Handbook of Biological Statistics (36). Genes with an FDR *p*-value below 0.05 were considered significantly enriched.

**FIG. 2.**
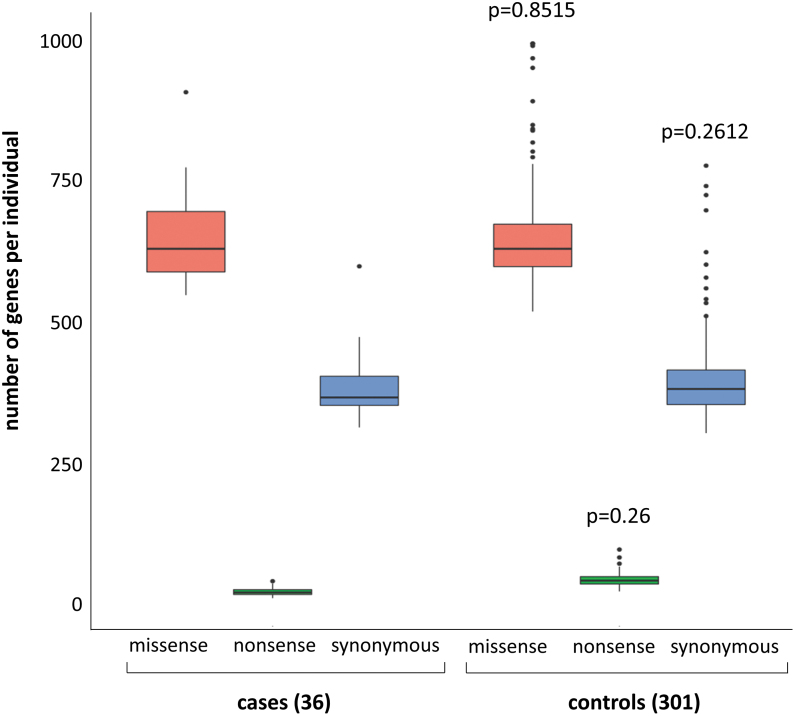
Box blot of rare SNVs in cases compared with controls with raw numbers of gene per individual with at least one rare SNV given as median and interquartile range, *p*-value of Mann–Whitney test. Nonsense variants included frameshift and stop codon variants. SNV, single-nucleotide variant.

As part of validating our bioinformatic pipeline variant calling, we compared, using a Mann–Whitney test, the unbiased gene burden of synonymous variants, presumptively mostly or all neutral. Similar burdens were observed in cases versus controls, as required to meaningfully interpret any differences observed for protein-altering, potentially pathogenic variants (Fig. 2).

### CH-related gene analysis

From our filtered exome data set, we extracted all the rare variants in 27 genes known to be associated with CH, and genes reported to be mutated in syndromes associated with TD (Supplementary Table S1) (8–19,37). Several tools were used to evaluate the pathogenicity of the variants, including SIFT, Polyphen, Mutation Taster, and CADD (available with ANNOVAR annotation). A rare variant was considered pathogenic if the CADD score was higher than 15 and at least one of the other pathogenicity scores classified it as deleterious or pathogenic. A rare variant was considered likely pathogenic if the CADD score was between 10 and 15 and at least one of the other pathogenicity scores classified it as deleterious or pathogenic. A rare variant was considered benign if the CADD score was under 10 and no more than one of the other pathogenicity scores classified it as deleterious or pathogenic.

Finally, a rare variant was considered of uncertain significance if the CADD score was between 10 and 20 and none of the other pathogenicity scores classified it as deleterious or pathogenic or if the CADD score was under 10 but at least one of the other pathogenicity scores classified it as deleterious or pathogenic. We only kept pathogenic and likely pathogenic variants for this analysis. We compared the number of cases and controls with pathogenic or likely pathogenic variants using a Fisher exact test.

### Sanger sequencing

Sanger sequencing using standard methods was carried out to validate variants in genes identified from the unbiased approach and in CH-related genes. Variants in CH-related genes were also assessed in the parents. Polymerase chain reaction products were sequenced using the 3730xl DNA Analyzer technology (Applied Biosystems, Foster City, CA) in the Genome Quebec Innovation Centre.

## Results

### CHTD cases are not enriched in rare protein-altering variants

WES analysis resulted in a total of 22,664 genes with at least one variant in a coding region and 16,383 genes with at least one rare variant (MAF <0.01 in public databases and frequency <0.01 in the control cohort) (Fig. 1). Of these, 8201 genes with at least one rare protein-altering variant were found in cases. As expected, the number of genes with synonymous variants was similar in cases and controls (*p*-value: 0.2612). More importantly, the number of genes with nonsense or missense variants was also similar between cases and controls (*p*-value of 0.26 for nonsense variants and 0.8515 for missense variants) (Fig. 2).

### Gene burden analysis does not reveal new candidate predisposing genes

**FIG. 3.**
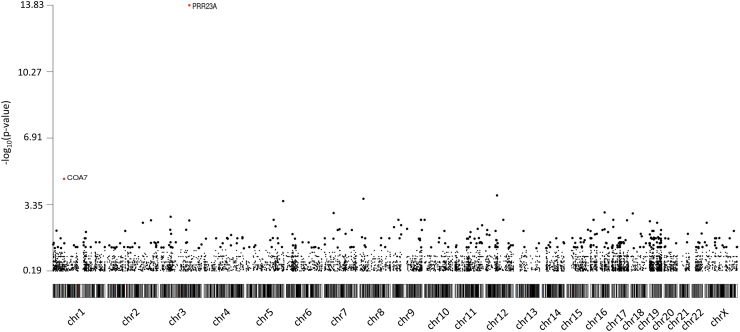
Manhattan plot of the gene-based burden test. The plot shows the negative log10 of the *p*-value of the Fisher exact test per chromosome. *PRR23A* had a FDR-corrected *p*-value of 1.41 × 10^–10^ and COA7 of 0.043. However, variants in these genes were false positives. FDR, false discovery rate.

We performed a gene-based burden analysis to identify genes enriched with rare protein-altering variants in the cases, using the 8201 genes (Fig. 3). A one-tailed Fisher exact test was conducted, and only genes with an odds ratio >1 were considered for the FDR correction. Only two genes were initially considered candidate genes, *PRR23A* and *COA7. PRR23A* had four protein-altering variants. However, this gene has two paralogs (*PRR23B* and *PRR23C*) with a duplication of a segmental region. Thus, variants identified in *PRR23A* are due to misalignment from the pipeline. *COA7* harbored one protein-altering variant in six patients, but Sanger sequencing revealed that the variant was a false positive.

### Identification of rare pathogenic or likely pathogenic variants in CH-related genes

**FIG. 4.**
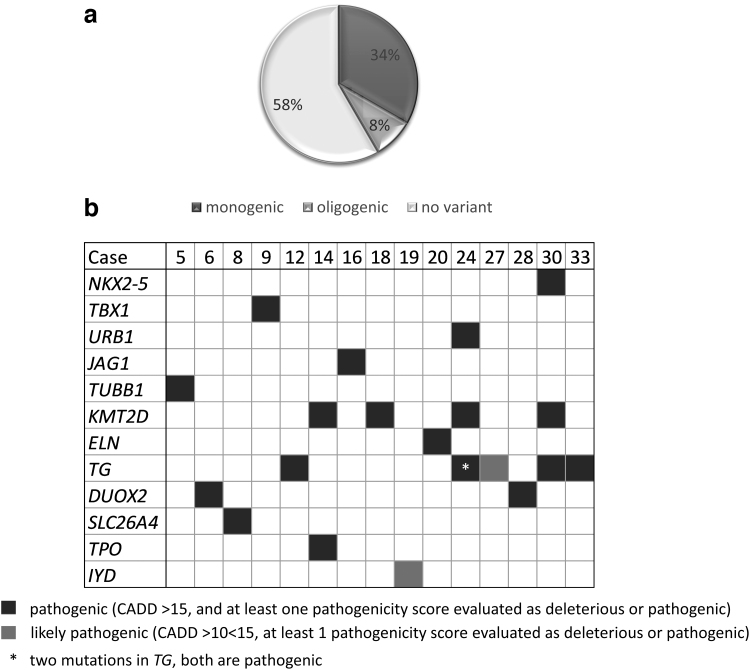
Burden of rare pathogenic or likely pathogenic variants detected in CHTD cases. (**a**) Rare pathogenic or likely pathogenic variants in CH-related genes were detected in 15 of 36 cases (42%). In these patients, three of them (8%) have at least one variant in more than one gene. (**b**) Rare pathogenic or likely pathogenic variants in CH-related genes per case. CH, congenital hypothyroidism.

We next identified all pathogenic and likely pathogenic variants in CH-related genes carried by NS-CHTD cases (Table 1). Twenty-seven genes related to a variable extent to TD or dyshormonogenesis (D) were assessed (Supplementary Table S1). The percentage of cases with pathogenic or likely pathogenic variants in CH-related genes (44%), before validation by Sanger sequencing, is similar to what was observed in the controls (49%) (*p*-value of 0.8761). Unfortunately, we did not have access to the controls' DNA to validate variants. In cases, all variants were validated by Sanger sequencing. Forty-two percent of CHTD cases (15/36) have at least one validated pathogenic or likely pathogenic variant and 8% (3/36) have at least one pathogenic or likely pathogenic variant in more than one CH-related gene (Fig. 4a). The most frequently mutated gene is *TG* (six mutations in five patients), followed by *KMT2D* (four mutations in four patients).

**Table 1.** Rare Pathogenic or Likely Pathogenic Variants Identified in Congenital Hypothyroidism-Related Genes in Nonsyndromic-Congenital Hypothyroidism Due to Thyroid Dysgenesis Cases

Patient	Gene	Variant position (GRCh37)	Amino acid change	Status	Inheritance	rs number	GnomAD MAF	In silico prediction			
								SIFT	Polyphen-2 HDIV	Mutation Taster	CADD score
5	TUBB1	20:57599401C>T	Arg370Cys	Het	Father	rs62639974	0.0042	Deleterious	Damaging	Tolerated	31
6	DUOX2	15:45392277C>T	Cys1052Tyr	Het	Father	rs76343591	0.0013	Deleterious	Benign	Tolerated	19
8	SLC26A4/Pendrin	7:107355874C>T	Arg776Cys	Het	Father	rs111033255	0.0018	Tolerated	Damaging	Deleterious	28.5
9	TBX1	22:19751796G>A	Val211Met	Het	U	rs749275495	2.848e-05	Deleterious	Damaging	Deleterious	29.2
12	TG	8:133919047G>T	Arg1250His	Het	Mother	rs114944116	0.0024	Deleterious	Possibly damaging	Tolerated	17.93
14	KMT2D	12:49424111G>A	His4651Tyr	Het	Father	rs767232021	4.406e-05	Deleterious	Benign	Tolerated	23
	TPO	2:1497783C>G	Gln660Glu	Het	Father	rs121908088	0.0003	Deleterious	Damaging	Deleterious	53
16	JAG1	20:10620426A>G	Phe1126Ser	Het	Mother	—	—	Deleterious	Damaging	Deleterious	28.3
18	KMT2D	12:49432365G>A	Ala2925Val	Het	Mother	rs199547661	0.0017	Deleterious	Benign	Tolerated	17.15
19	IYD	6:150716673G>A	Cys257Tyr	Het	Mother	rs115446362	0.0024	Deleterious	Benign	Tolerated	10.42
20	ELN	7:73482987G>A	Gly711Asp	Het	Mother	rs41511151	0.003	Tolerated	Damaging	Tolerated	26.3
24	TG	8:133895162G>C	Gln331His	Het	Father	rs61745783	0.0003	Deleterious	Damaging	Tolerated	19.45
	TG	8:133984047A>G	Glu1995Gly	Het	Mother	rs190914906	0.0007	Deleterious	Damaging	Tolerated	27.7
	URB1	21:33697576G>A	Ser 1695Leu	Het	Father	rs187640762	0.0069	Tolerated	Damaging	Tolerated	39
	KMT2D	12:49424759C>T	Asp4530Asn	Het	Mother	rs768143170	3.249e-05	Deleterious	Possibly damaging	Tolerated	27.7
27	TG	8:133883643A>G	Ile109Val	Het	Mother	rs35301433	0.004	Deleterious	Benign	Tolerated	13.35
28	DUOX2	15:45393425TGAAC>T	Ser965fsX994	Het	Mother	rs530719719	0.003	—	—	—	—
30	NKX2–5	5:172659915G>A	Pro211Leu	Het	Father	rs3729754	0.0002	Tolerated	Possibly damaging	Tolerated	18.77
	KMT2D	12:49428694T>C	Asp3419Gly	Het	Mother	rs146044282	0.0016	Deleterious	Damaging	Deleterious	29.2
	TG	8:133894816G>A	Arg283Leu	Het	Mother	rs146926250	0.0008	Deleterious	Damaging	Deleterious	31
33	TG	8:133953740A>C	Asp1729Ala	Het	Father	rs61744749	0.0061	Deleterious	Benign	Tolerated	23

het, heterozygous; U, unknown; MAF, minor allele frequency.

The patients with variants in *JAG1* and *NKX2-5* have an ectopy, but neither cardiac nor pulmonary anomalies. The patient with a *TUBB1* variant also has an ectopy but her hematological parameters were not assessed. Of note, the affected mother and daughter do not share variants in either of the genes associated with CHTD or in interesting candidate genes.

Moreover, 28% of cases (10/36) have at least one variant in dyshormonogenesis-related genes (Fig. 4b). Of note, when we compared thyrotropin (TSH) and thyroxine, cases with variants in those genes did not have more severe hypothyroidism than cases without (Table 2 and Supplementary Table S2). Finally, we assessed if patients with variants in more than one gene (oligogenic group) had a more severe phenotype than cases with variants in only one gene (monogenic group), as shown by Yamaguchi *et al.* (38). However, no difference was observed in the biochemical severity of CH between our oligogenic and monogenic groups (Table 3 and Supplementary Table S2).

**Table 2.** Comparison of Congenital Hypothyroidism Severity Between Cases with Variant in Dyshormonogenesis-Related Genes and Cases Without Variant in Dyshormonogenesis-Related Genes

	Cases with variants in D-related genes (*N* = 9)	Cases without variants in D-related genes (*N* = 22)	*p*
NBS-TSH (mU/L)	138 (21–217)	133 (21–281)	0.7627
NBS-TT4 (nmol/L)	114.5 (47–263)	74 (16–194)	0.3246
Diagnostic-TSH (mU/L)	51.12 (14.09–310)	257.8 (5–714.1)	0.1516
Diagnostic-fT4 (pmol/L)	8.16 (2.9–23.16)	6.61 (0.4–15.58)	0.4327
Diagnostic-T3 (nmol/L)	2.3 (1.2–3.1)	1.55 (0.3–3.1)	0.2348

D, dyshormonogenesis; fT4, free thyroxine; *N*, number of cases; NBS, newborn screening; T3, triiodothyronine; TSH, thyroid stimulating hormone; TT4, total thyroxine.

**Table 3.** Comparison of Congenital Hypothyroidism Severity Between Monogenic and Oligogenic Groups

	Monogenic (*N* = 10)	Oligogenic (*N* = 3)	*p*
NBS-TSH (mU/L)	93.5 (22–211)	140 (21–217)	0.8112
NBS-TT4 (nmol/L)	111 (27–263)	85 (62–144)	>0.9999
Diagnostic-TSH (mU/L)	186.95 (14.09–444)	100 (22.5–157.61)	0.4818
Diagnostic-fT4 (pmol/L)	6.78 (2.9–23.16)	8.16 (3.24–12.4)	>0.9999
Diagnostic-T3 (nmol/L)	1.6 (0.3–3.1)	2.3 (1.2–2.8)	0.5545

## Discussion

The molecular cause of NS-CHTD with ectopic thyroid or athyreosis remains elusive. To our knowledge, this is the first WES study performed on well-characterized TD patients (specifically ectopic thyroid or athyreosis documented by technetium scintigraphy). We evaluated the burden of rare protein-altering variants in NS-CHTD using a case–control design (26). As shown in Figure 2, NS-CHTD cases are not enriched in rare missense and nonsense variants compared with a control population of similar ethnicity.

We used an unbiased approach to identify new potential predisposing genes. After correction for misalignment and validation with Sanger sequencing, no gene was identified. In addition, we looked for rare variants in genes known to be associated with CH. The percentage of cases and controls who had at least one pathogenic or likely pathogenic variant identified by WES was similar. This confirms that heterozygous variants are not sufficient to cause CHTD. Validated pathogenic or likely pathogenic variants in CH-related genes were identified in 42% of our CHTD cases, which is comparable with the literature (Supplementary Tables S3 and S4) (39). Thus, it suggests that unraveling the genetic component of CHTD would require more than increasing the number of genes assessed.

Of note, most other studies did not report scintigraphy to identify an ectopic thyroid. One study, in a Saudi Arabian cohort, identified causative variants in 44% (11/25) of the cases with TD (40). However, in that study, when cases with *TSHR* variants, a gene associated with hypoplasia or apparent athyreosis but neither with ectopy nor with true athyreosis (41,42), were excluded, the discovery rate drops to 16%. Two other studies used targeted NGS to identify variants in CH-related genes in cases with CHTD (38,43).

In the Japanese cohort (38), 18/32 (56%) of CHTD cases had a variant in genes associated with CHTD and dyshormonogenesis, but only 7/32 (22%) had a pathogenic or likely pathogenic variant, the majority being of unknown significance (38). In the Italian cohort (43), 83 out of 177 (46.9%) were diagnosed as CHTD (43). Thirty-nine CHTD cases (47%) had a variant that passed the author's pathogenicity criteria. However, after removing the syndromic cases, to compare with our NS cohort, the discovery rate in the Italian NS-CHTD cases is 21%. Almost all variants identified in our study were heterozygous and inherited from a healthy parent, which suggests that these variants have only a predisposing role and supports our two-hit model.

Studies have recently shown that patients with TD have variants in genes related to dyshormonogenesis, contributing to their hypothyroidism. In our cohort, patients with variants in dyshormonogenesis-related genes were not more severely affected than those without such variants (Table 2). It is important to mention that even though such variants may affect the severity of CH, they cannot explain the TD.

Oligogenicity has been proposed as a model to explain CHTD, although it is not compatible with the observed discordance between MZ twins. Eight percent of our cases had oligogenic variants, consistent with recent studies (Supplementary Table S3) (38,43). Yamaguchi *et al.* suggested that oligogenic cases have more severe hypothyroidism than monogenic cases, since they had a higher TSH level at the first visit (38), but this was not observed in our cohort (Table 3).

As observed in other studies, variants identified in CHTD cases do not always segregate with the phenotype (38,41,43). For instance, one case has two variants shared by her healthy father (case 14) and another has two variants shared by her healthy mother (case 30). Moreover, one patient has four variants, two inherited from the unaffected mother and two from the unaffected father. Finally, one case with a *KMT2D* variant is an MZ twin whose unaffected twin and mother also carry this variant (case 18). These results support the incomplete penetrance proposed by others to explain CHTD (5). They could also support our two-hit hypothesis, suggesting that the occurrence of CHTD requires a genetic predisposition, and also an epigenetic mechanism, monoallelic expression, or an early postzygotic mutation, as proposed by others (44).

In summary, this is the first study assessing the burden of rare protein-altering variants in well-defined NS-CHTD cases. We did not observe enrichment in rare protein-altering variants in cases compared with controls. This might be due to several reasons: the small number of cases in our cohort limits our statistical power. For NS complex diseases, the number of patients needed to identify candidate genes with sufficient statistical power needs to be much higher, probably more than a thousand cases (45–47).

Alternatively, causal variants for CHTD might, for mechanistic reasons, lie preferentially in nonprotein coding genomic regions, such as transcriptional regulatory sequences, which are not assessed in exome sequencing studies. Finally, it might also mean that in nonconsanguineous NS-CHTD patients, genetics is not a major factor contributing to CHTD, although this leaves the issue of the observed 40-fold increase in relative risk in first-degree relatives unresolved. Important unidentified environmental factors may possibly be involved. Recently, Wassner (48) suggested that broad-based testing of thyroid-related genes in CH cases is probably not efficient and clinically useful, particularly for NS-CHTD.

On the contrary, we identified pathogenic or likely pathogenic variants in CH-related genes in 42% of the cases. This is comparable with what is found in other studies (38,40,43). Nonetheless, considering the absence of segregation between the genotype and the phenotype in many cases, those results are unlikely to be useful for patient counseling, as suggested by Wassner for several variants identified in CH patients (48). Moreover, even though variants in genes related to dyshormonogenesis can explain the CH, they cannot explain the TD, as CH and TD are two distinct phenotypes.

Thus, to identify “thyroid dysgenesis-specific” genes, we might need cases with TD but without CH, which is an extremely rare occurrence considering that most TD cases are identified through screening for CH. Other diseases with stronger evidence for a genetic component, such as autism spectrum disorder (ASD), encountered several challenges to identify ASD-specific genes despite having access to larger cohorts than for NS-CHTD (49,50). Therefore, identifying the genetic component will require much larger and well-phenotyped cohorts, an elusive task considering the relatively low incidence of CHTD.

One option to unravel TD etiology might be to sequence a higher number (than what was already attempted (24)) of MZ twins discordant for CHTD, hoping to find a genetic explanation for their phenotypic difference. Omics studies (genomic, epigenomic, and transcriptomic studies) on several ectopic thyroid tissues are another promising approach. However, many tissues would be required since, to date, studies that attempted these approaches have been unsuccessful (22,23,51,52).

In conclusion, a major role for genetics in CHTD remains unproven. Alternative approaches, such as whole-genome sequencing and epigenomics, might allow for a better understanding of the causes of TD, if sufficiently large case cohorts can be analyzed to have adequate statistical power. Alternatively, basic research to identify the mechanisms underlying thyroid relocalization during embryogenesis (53) might lead to the identification of new genes implicated in this process. Variants in these genes could then be assessed in the existing CHTD cohorts.

## Supplementary Material

Supplemental data

Supplemental data

Supplemental data

Supplemental data

Supplemental data
