# Supplementary material for: Whole-Exome Sequencing in Congenital Hypothyroidism Due to Thyroid Dysgenesis
Source: Thyroid. 2022 May 17;32(5):486–95. doi: 10.1089/thy.2021.0597 (PMC9145262; doi:10.1089/thy.2021.0597)

Figure S1: PCA plot of eigenvalues comparing the cases, controls, and 1000genome samples. Comparison of the eigen-value PCA plot of the cases data versus the controls and the general population of 1000genome’s (1KG) data. Most cases and controls belong to the Caucasian ancestry cluster (red circle) or the admixed Americans ancestry clusters (green ellipses). We have removed nine controls out of 310 according to the stratification analysis. The 1KG population samples are represented by a purple empty circle, our control samples by a black full circle and the cases by a green full triangle. The removed samples are indicated in the dash-lined boxes.


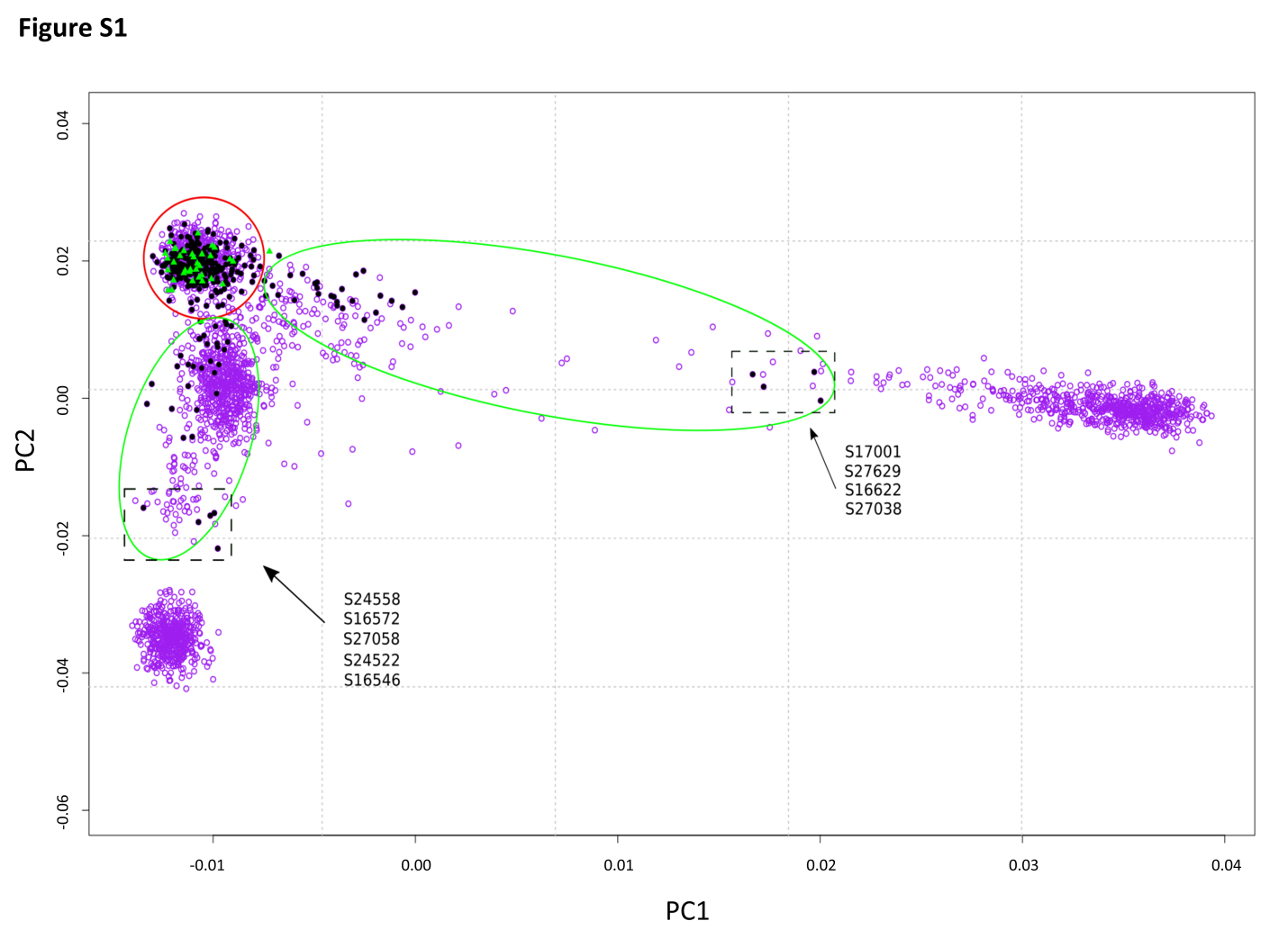

Supplement: Supplemental data [file Suppl_FigS1.docx]
