# Supplementary material for: Whole-Exome Sequencing in Congenital Hypothyroidism Due to Thyroid Dysgenesis
Source: Thyroid. 2022 May 17;32(5):486–95. doi: 10.1089/thy.2021.0597 (PMC9145262; doi:10.1089/thy.2021.0597)
Supplement: Supplemental data [file Suppl_TableS1.docx]

Table S1: List of CH-related genes

| gene name | gene ID |
| --- | --- |
| *FOXE1/TTF2* | 2304 |
| *GLIS3* | 169792 |
| *NKX2-1/TTF1* | 7080 |
| *NKX2-5* | 1482 |
| *PAX8* | 7849 |
| *SALL1* | 6299 |
| *TBX1* | 6899 |
| *URB1* | 9875 |
| *NTN1* | 9423 |
| *JAG1* | 182 |
| *CDCA8/BOREALIN* | 101738843 |
| *TUBB1* | 81027 |
| *DYRK1A* | 1859 |
| *ELN* | 2006 |
| *KMT2D/MLL2* | 8085 |
| *KDM6A* | 7403 |
| *KAT6B* | 23522 |
| *GNAS* | 2778 |
| *TSHR* | 7253 |
| *SLC5A5/NIS* | 6528 |
| *TPO* | 7173 |
| *DUOX2* | 50506 |
| *DUOXA2* | 405753 |
| *SLC26A4 (Pendrin)* | 5172 |
| *TG* | 7038 |
| *IYD/DEHAL1* | 389434 |
| *SLC26A7* | 115111 |

CH: congenital hypothyroidism
