# Supplementary material for: Whole-Exome Sequencing in Congenital Hypothyroidism Due to Thyroid Dysgenesis
Source: Thyroid. 2022 May 17;32(5):486–95. doi: 10.1089/thy.2021.0597 (PMC9145262; doi:10.1089/thy.2021.0597)
Supplement: Supplemental data [file Suppl_TableS2.docx]

Table S2: Clinical description of NS-CHTD cases

| Patients | Sex | Phenotype | Age at screening (days) | Screening | | Age at diagnosis | Diagnosis | | | |
| --- | --- | --- | --- | --- | --- | --- | --- | --- | --- | --- |
|  |  |  |  | TSH (mU/L) | Tot T_4_ (nmol/L) |  | TSH (mU/L) | fT_4_ (pmol/L) | T_3_ (nmol/L) |  |
| 1 | F | E | NA | NA | NA | 14 days | 261 | NA | 3.1 |  |
| 2 | M | E | 1 | 21 | 112 | 14 days | 28.89 | 11.66 | 1.5 |  |
| 3* | F | E | 3 | 37 | 112 | 8 years | 6.61 | 8.47 | NA |  |
| 4 | M | A | 2 | 154 | 16 | 10 days | 714.07 | 3.54 | 1.1 |  |
| 5 | F | E | 2 | 24 | 136 | 20 days | 52.69 | 9.17 | NA |  |
| 6 | F | E | 2 | 54 | 263 | 7 days | 14.09 | 23.16 | 2.3 |  |
| 7 | F | E | 1 | 104 | 88 | 13 days | 181.21 | 6.97 | 1.6 |  |
| 8 | M | E | 1 | 48 | 151 | 10 days | 51.12 | 12.34 | 2.2 |  |
| 9 | F | E | NA | NA | NA | NA | NA | NA | NA |  |
| 10 | F | E | NA | NA | NA | NA | NA | NA | NA |  |
| 11 | F | E | NA | 150 | NA | 8 days | 257.75 | 4.38 | 1.3 |  |
| 12 | F | E | 2 | 29 | 190 | 33 days | 23.01 | 10.59 | 3.1 |  |
| 13 | F | E | 2 | 138 | 142 | 13 days | 112.21 | 7.98 | 2.8 |  |
| 14 | F | E | 1 | 217 | 62 | 8 days | >100 | 3.24 | 1.2 |  |
| 15 | F | A | 1 | 281 | 27 | 9 days | >100 | <1.9 | 0.9 |  |
| 16 | M | E | 1 | 171 | 111 | 13 days | 186.95 | 6.25 | 1.6 |  |
| 17 | F | E | 2 | 28 | 74 | 16 days | 333.98 | 2.44 | 1.0 |  |
| 18 | M | A | 4 | 22 | 71 | 15 days | 444 | NA | <0.3 |  |
| 19 | F | A | NA | NA | NA | NA | NA | NA | NA |  |
| 20 | F | E | 2 | 133 | 48 | 10 days | 414.00 | 3.66 | 1.0 |  |
| 21 | F | E | NA | NA | NA | NA | NA | NA | NA |  |
| 22 | F | E | NA | NA | NA | NA | NA | NA | NA |  |
| 23 | F | E | 2 | 237 | 70 | 42 days | 442.71 | 5.9 | 1.8 |  |
| 24 | F | E | 3 | 140 | 85 | 10 days | 157.61 | 8.16 | 2.3 |  |
| 25 | F | E | NA | NA | NA | NA | 5.0 | 9.24 | 2.3 |  |
| 26 | F | E | 1 | 34 | 194 | 10 days | 9.26 | 15.58 | 2.5 |  |
| 27 | F | E | NA | 211 | 57 | 15 days | >100 | 2,9 | 1,3 |  |
| 28 | M | E | NA | 141 | 47 | 14 days | 310 | 3,4 | 1,3 |  |
| 29 | F | E | 2 | 65 | 102 | 10 days | 177.00 | 9,78 | 2.6 |  |
| 30 | F | E | 3 | 21 | 144 | 14 days | 22.5 | 12,4 | 2.8 |  |
| 31 | F | E | 2 | 149 | 63 | 18 days | 284 | 12.6 | 1,6 |  |
| 32 | F | E | NA | 81 | NA | NA | NA | NA | NA |  |
| 33 | F | E | NA | 138 | NA | 14 days | 253 | 6.78 | 2.3 |  |
| 34 | F | E | 2 | 236 | NA | 12 days | 592 | 2,6 | 1,4 |  |
| 35 | M | E | NA | 177 | NA | 10 days | >100 | 2,7 | 1,3 |  |
| 36 | F | E | NA | 103 | 38 | 16 days | 295 | 0,4 | NA |  |

A: athyreosis, E: ectopy, F: female, fT_4_: free thyroxine, M: male, T_3_: triiodothyronine, Tot T_4_: total thyroxine, TSH: thyroid stimulating hormone, TG: thyroglobulin, NA: not available, * patient’s details in Stoppa-Vaucher et al., JCEM, 2010 (25)
