# Supplementary material for: Whole-Exome Sequencing in Congenital Hypothyroidism Due to Thyroid Dysgenesis
Source: Thyroid. 2022 May 17;32(5):486–95. doi: 10.1089/thy.2021.0597 (PMC9145262; doi:10.1089/thy.2021.0597)
Supplement: Supplemental data [file Suppl_TableS3.docx]

Table S3: Literature review of articles that include CHTD cases

|  | de Filippis, Hum Mol Genet, 2017 (43) | Zou, JCEM, 2018 (40) | Yamaguchi, JCEM, 2020 (38) |
| --- | --- | --- | --- |
| Sequencing approach | TNGS | WES | TNGS |
| Genes studied | *NKX2-1, PAX8 FOXE1, GLIS3, JAG1, TSHR, SLC26A4, TG, TPO, DUOX2, DUOXA2*  (11 in total) | NA | *NKX2-1, NKX2-5, FOXE1, HHEX, GLIS3, TSHR, JAG1, TG, TPO, DUOX2, DUOXA2, DUOX1, DUOXA1, SLC5A5, SLC26A4, IYD, SLC16A2, SECISBP2, GNAS, DIO1, DIO2, DIO3, CDCA8*  (24 in total) |
| Number of CHTD patients | 83 | 25 | 32 |
| Ectopic thyroid | 30 | NA | NA |
| Athyreosis | 23 | NA | NA |
| Type of variants assessed | nonsense, frameshift, splice site, missense; disruptive variant: deleterious in 5/7 algorithms of the dbSNP database | VUS: considered mutations if <0.01, damaging or disease causing in 3/4 prediction tools, strict segregation; diseases causing mutation: biallelic variants | variants called benign or likely benign were excluded |
| *In silico* prediction tools | SIFT, Polyphen2, Mutation Taster, Mutation Assessor, LRT, FATHMM; for intronic variants: NetGene2v.2.4 ESEfinder2.0, BDGP | Mutation Taster, PolyPhen-2, SIFT, PROVEAN | ACMG guidelines |
| % of variants identified in CHTD cases | 47% for all CHTD cases  21% after removing syndromic CHTD | 44% for all CHTD cases 20% after removing cases with *TSHR* variant: | 56% for all variant, 22% for pathogenic or likely pathogenic variants |
| % of CHTD cases in oligogenic group | 10% | 12.5% | NA |

CHTD: congenital hypothyroidism due to thyroid dysgenesis, TNGS: targeted next-generation sequencing, WES: whole exome sequencing, NA: Not available
