# Supplementary material for: Whole-Exome Sequencing in Congenital Hypothyroidism Due to Thyroid Dysgenesis
Source: Thyroid. 2022 May 17;32(5):486–95. doi: 10.1089/thy.2021.0597 (PMC9145262; doi:10.1089/thy.2021.0597)
Supplement: Supplemental data [file Suppl_TableS4.docx]

Table S4: Comparison between the percentage of cases with variants in CH-related genes of our NS-CHTD cohort and in published cohorts

|  | Percentage of cases with a variant in CH-related genes (cases/total cases) | Two-tailed Fisher exact test, p-value | 95% Confidence interval | |
| --- | --- | --- | --- | --- |
|  |  |  | Lower limit | Upper limit |
| Present cohort | 42 (15/36) |  |  |  |
| de Flippis, 2017 | 21 (17/83) | 0.0934 | 02061 | 1.882 |
| Zou, 2018 | 16 (4/25) | 0.1719 | 0.0838 | 1.4164 |
| Yamaguchi, 2020 | 22 (7/32) | 0.2285 | 0.1609 | 1.5917 |
